# Preliminary Evidence That Anodal Transcranial Direct Current Stimulation Enhances Time to Task Failure of a Sustained Submaximal Contraction

**DOI:** 10.1371/journal.pone.0081418

**Published:** 2013-12-09

**Authors:** Petra S. Williams, Richard L. Hoffman, Brian C. Clark

**Affiliations:** 1 Ohio Musculoskeletal and Neurological Institute, Ohio University, Athens, Ohio, United States of America; 2 Department of Physical Therapy and Athletic Training, Northern Arizona University, Flagstaff, Arizona, United States of America; 3 Department of Biomedical Sciences, Ohio University, Athens, Ohio, United States of America; 4 Department of Geriatric Medicine and Gerontology, Ohio University, Athens, Ohio, United States of America; The University of Queensland, Australia

## Abstract

The purpose of this study was to determine whether anodal transcranial direct current stimulation (tDCS) delivered while performing a sustained submaximal contraction would increase time to task failure (TTF) compared to sham stimulation. Healthy volunteers (n = 18) performed two fatiguing contractions at 20% of maximum strength with the elbow flexors on separate occasions. During fatigue task performance, either anodal or sham stimulation was delivered to the motor cortex for up to 20 minutes. Transcranial magnetic stimulation (TMS) was used to assess changes in cortical excitability during stimulation. There was no systematic effect of the anodal tDCS stimulation on TTF for the entire subject set (n = 18; *p* = 0.64). Accordingly, *a posteriori* subjects were divided into two tDCS-time groups: Full-Time (n = 8), where TTF occurred prior to the termination of tDCS, and Part-Time (n = 10), where TTF extended after tDCS terminated. The TTF for the Full-Time group was 31% longer with anodal tDCS compared to sham (*p* = 0.04), whereas TTF for the Part-Time group did not differ (*p* = 0.81). Therefore, the remainder of our analysis addressed the Full-Time group. With anodal tDCS, the amount of muscle fatigue was 6% greater at task failure (*p* = 0.05) and the amount of time the Full-Time group performed the task at an RPE between 8–10 (“very hard”) increased by 38% (*p* = 0.04) compared to sham. There was no difference in measures of cortical excitability between stimulation conditions (*p* = 0.90). That the targeted delivery of anodal tDCS during task performance both increased TTF and the amount of muscle fatigue in a subset of subjects suggests that augmenting cortical excitability with tDCS enhanced descending drive to the spinal motorpool to recruit more motor units. The results also suggest that the application of tDCS during performance of fatiguing activity has the potential to bolster the capacity to exercise under conditions required to derive benefits due to overload.

## Introduction

In healthy individuals, fatigue is both a physical and perceptual experience that is a normal and expected physiologic reaction to sustained and intense activity [1]–[3]. The physical experience of fatigue involves observable decrements in task performance over time, such as a decline in force output or task accuracy; whereas the concurrent perceptual experience refers to the increased sense of effort required to sustain task performance [1]–[6]. Fatigue is a multi-factorial physiologic process involving continual functional adjustments in the nervous system and the muscle throughout the contraction [4], [7]. The major question in fatigue studies for the past three decades has been “which of these events determine performance and which are simply incidental by-products” as “finding those which are not responsible is as valuable as investigating those that are” [7,p. 693]. This question is driven by the assumption that interventions could be developed to target the limiting physiologic mechanisms thereby reducing fatigue and prolonging performance.

While the mechanisms of fatigue are generally accepted to be task specific with no one single cause [1], [2], [7], [8], it has been shown that the nervous system’s failure to maintain sufficient activation of the muscle is a significant contributor to task failure in sustained submaximal contractions [2], [4], [9]–[11], particularly for low intensity contractions (e.g., <30% of maximal strength) [12]. Recent work has provided convincing evidence that as task duration progresses, spinal excitability declines as the motorneurons become progressively resistant to activation [13]–[15]. Thus, in order to sustain task performance, the amount of excitatory descending drive from supraspinal regions increases to compensate for the reduced excitability of the spinal region [2], [4], [11], [16], [17]. However, despite the compensatory mechanisms from supraspinal inputs, task failure remains inevitable [14], [15], [18]. This suggests that task duration may be prolonged if supraspinal excitability could be specifically manipulated during task performance.

In the past two decades transcranial direct current stimulation (tDCS), a form of non-invasive focal neurostimulation using weak direct electrical currents (1–2 mA) delivered via sponge electrodes (25–35 cm^2^) placed on the scalp, has become a major tool to investigate the cortical mechanisms of neural plasticity and motor learning because of its capacity to elicit sustained but transient changes in cortical excitability combined with a relative ease in delivery and utility for sham controlled double-blind experiments [19]–[25]. Unlike transcranial magnetic stimulation (TMS), the current does not directly stimulate axons causing them to discharge [26], instead tDCS acutely modulates the resting membrane potential (i.e., subthreshold depolarization or hyperpolarization) of the tissue under the electrodes thereby adjusting the ongoing neuronal firing activity [19], [24], [27], [28]. Numerous studies have demonstrated that anodal tDCS, where current flows from anode over the motor cortex to cathode over the contralateral forehead, acutely increases motor cortex excitability as measured by TMS after the stimulation period [26]–[32]. The degree and duration of the after-stimulation effects of tDCS on cortical excitability are known to depend upon the dosage of current delivered, whereas the effect on motor learning is also influenced by the timing of stimulation (i.e., before or during) relative to motor practice [25], [33]. Anodal tDCS delivered for a minimum of 13 minutes (2.0 mA, 35 cm^2^ electrodes) while the subject is at rest has been shown to increase measures of intracortical facilitation and simultaneously decrease intracortical inhibition for up to 90-minutes after the stimulation [26], [28], [30], [34], [35]. In healthy subjects and individuals after stroke, anodal tDCS (1.0–2.0 mA, 25–35 cm^2^ electrodes, 10–20 minutes) applied over the motor cortex *during* task practice, relative to anodal tDCS delivered *prior* to task practice as a pre-conditioning treatment, has been shown to improve the speed and accuracy of motor performance, the rate of learning new motor tasks, and the recovery of function [23]–[25], [28], [32], [33], [36]–[38]. Despite these findings, the direct relationship between tDCS induced changes in motor performance and cortical excitability remain largely unexplored as the majority of studies evaluating tDCS rarely examine both outcomes in the same experiment [32], [33], [38].

The capacity of anodal tDCS to both increase cortical excitability and improve motor performance suggests that anodal tDCS also has the potential to prolong muscle endurance during a sustained submaximal fatiguing contraction as well as to further the mechanistic study of fatigue. Unlike pharmacologic manipulation with stimulants like caffeine that have a systemic effect on all levels on the neuromuscular system [39] the effects of tDCS have been shown to be localized to the tissue underneath the electrodes [28], [40]–[42] thereby providing a targeted experiment strategy to modulate supraspinal excitability. Presently, two studies have examined the effects of tDCS on fatigue task duration with mixed results [43], [44]. In these studies, subjects completed two submaximal fatiguing contractions during the same test session and either anodal (excitatory 1.5 mA–2 mA, 10 min, 25–35 cm^2^), cathodal (inhibitory) or sham tDCS was used as a pre-conditioning treatment delivered to resting subjects *prior* to the second contraction. Only one study found a systematic shorter task duration for the second relative to the first contraction in the session [43], and also reported a significantly longer second contraction time with anodal tDCS delivered *before* the second contraction compared to both cathodal and sham tDCS whereas the other study found no differences in contraction times [44]. Taken together with the data from motor learning studies regarding the enhanced efficacy of tDCS when delivered *during* task practice, these studies suggest that the potential for anodal tDCS to modulate supraspinal excitability in a manner that will prolong task duration during sustained submaximal contractions may be better assessed if the focal brain stimulation were delivered *while performing* the fatigue task.

Therefore, the purpose of this study was to determine whether anodal tDCS delivered *while performing* a sustained submaximal elbow flexion task to failure would increase task duration when compared to sham tDCS. We hypothesized that anodal tDCS would prolong the time to task failure (TTF) relative to sham stimulation. To explore the direct relationship between changes in cortical excitability and motor performance we used single pulse TMS (i.e., motor evoked potential MEP amplitude) to measure the neurophysiologic effects of tDCS during fatigue task performance.

## Methods

### Subjects

Eighteen healthy, right-handed, adult subjects (25±6 years; 9 men and 9 women) participated in the two testing sessions. Prior to participation, each subject attended an orientation session where they completed a series of questionnaires to confirm they were free from any known neurologic disorder, cardiovascular disease, or musculoskeletal injury in the upper extremities. Subjects identified themselves as highly active (n = 3, 1 male, 2 female,) moderately active (n = 6, 4 male, 2 female), or low active (n = 9, 4 male, 5 female) based on the Lipid Research Clinics Physical Activity Questionnaire [45], but denied participating in resistance training in the prior 3-months. Handedness was evaluated using the Edinburgh Handedness Inventory (80±22%) with scores greater than 40% indicating right hand dominance [46]. Subjects were also familiarized to the activities of the experiment by practicing maximum voluntary contractions (MVC) and the 20% MVC force-matching task used during the fatiguing contraction with the elbow flexors.

### Ethics Statement

The Institutional Review Board at Ohio University approved the study protocol, and all study participants provided written informed consent.

### General Overview of the Experiment Protocol and Testing Sessions

Subjects completed two experimental sessions separated by a minimum of 7 days (range: 7–13 days; average: 7.61±1.46 days). Both visits were conducted at the same time of day for each subject. During each test session, subjects performed one sustained submaximal isometric contraction equal to 20% of their MVC with the elbow flexors of the non-dominant arm (I.e. left arm) to task failure. Subjects were provided with visual feedback of their force output relative to the target force throughout the contraction. Either anodal tDCS stimulation or sham stimulation was delivered to the motor cortex for up to 20 minutes while the subjects performed the fatiguing contraction. Previous studies have found that the TTF can increase across sessions in certain individuals [47], therefore, subjects were randomly assigned to a counterbalanced order of stimulation conditions (Anodal visit-1→Sham visit-2∶3 men and 5 women; Sham visit-1→Anodal visit-2∶6 men and 4 women). Outcomes included the TTF, muscle fatigue which was quantified as the difference in elbow flexor MVC before vs. after the fatiguing contraction, rating of perceived exertion (RPE) using the modified Borg 10-point scale [48], and MEP amplitude. Both the subjects and the primary experimenter (PSW) were blinded to the tDCS stimulation condition and subjects were not informed of their TTF until both visits were completed.

**Figure 1:**
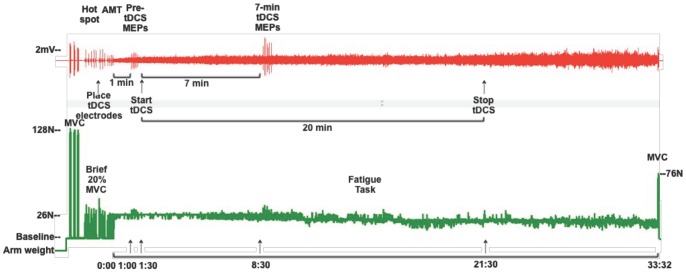
Experimental protocol. (*Top*) Representative traces of biceps electromyogram (EMG) and *(Bottom)* force signal from one subject during anodal tDCS. Resting arm weight was measured and added as a constant to the force signal to create a 0.0N baseline with the arm relaxed. Three elbow flexion maximum voluntary contractions (MVCs) were followed by a series of brief (3–5 sec) 20%MVC contractions during which motor evoked potentials (MEPs) were measured in the biceps EMG. After locating the biceps motor hotspot, but prior to determining active motor threshold (AMT), the transcranial direct current (tDCS) electrodes were placed on the scalp. One minute after starting the fatigue task, 6 MEPs were evoked over 30 seconds (Pre-tDCS MEPs), immediately after which the anodal tDCS was initiated (at time 1∶30). The second set of 6 MEPs were evoked at time 8∶30 of the fatigue task which was after 7 minutes of tDCS (7-min tDCS MEPs). At time 21∶30 of the fatigue task, the tDCS stimulation was terminated as the maximum duration for the tDCS protocol was 20 minutes. This subject’s time to task failure was 33∶32 minutes. At task failure, one final MVC was performed prior to relaxing.

Each test session started with testing elbow flexor MVC. Subjects then performed 2 bouts of short duration (3–5 second) non-fatiguing 20% MVC contractions during which the motor hotspot for the biceps brachii was located using TMS and the tDCS electrodes were placed. Subjects began the fatigue task with no tDCS (anodal or sham) being delivered. At 1-minute into the fatigue task, RPE was obtained and 6 motor evoked potentials were elicited using TMS (5-sec between pulses). Immediately after the TMS measures were completed, the tDCS stimulation was initiated (i.e., after 1-min 30-sec of the fatigue task). After 7 minutes of tDCS (at 8.5 minutes into the fatigue task), subjects were again asked their RPE and another 6 single TMS pulses were delivered. This time frame was chosen for two reasons: 1) it was anticipated that all subjects would be able to sustain the 20% MVC contraction at least 8-min 30-sec (and all subjects were able to perform the task through this point) and 2) anodal tDCS delivered for 7 minutes has been shown to be sufficient to modify cortical excitability with changes that persist for 5–10 minutes after turning off the current [26], [30], [31]. Regardless of the subject’s fatigue task contraction time, the tDCS stimulator was programmed to deliver current for a maximum of 20 minutes (anodal or sham), consistent with maximum stimulation durations currently reported in the literature examining motor function [33], [38]. Knowledge about the safety boundaries for current density and duration is limited; therefore, it is recommended that tDCS current density and duration stay within those used in already tested protocols [32], [38], [49]. Therefore, it was expected that the contraction time for some subjects would exceed the tDCS stimulation time. Subjects were not informed as to the timing for the tDCS (i.e. start time or duration) or when the RPE would be obtained. Just prior to task failure, subjects performed a maximal contraction (prior to relaxing), and a final RPE was obtained. See **Figure 1** for representative traces of EMG and force output recorded from one subject during the experimental protocol.

### Experimental Setup and Mechanical Recordings (Figure 2)

Subjects were seated upright in an adjustable chair with the left arm positioned next to the body in 10–15° of abduction and the upper arm supported at the elbow (Biodex System 4, Biodex Medical Systems Inc., Shirley, NY). The elbow joint was flexed to 90° and aligned with the axis of rotation of the torque motor with the forearm positioned in neutral (0° rotation) and the thumb pointing towards the ceiling. To obviate the use of the hand and wrist muscles during testing as well as to provide a secure and consistent attachment to the lever arm from the torque motor, subjects wore a prefabricated Wrist-Hand-Thumb orthosis (Model 1000, Orthomerica, Newport Beach, CA). The lever arm length was adjusted so that the point of application for the resistance was located just proximal to the wrist joint on the superior surface of the forearm. The orthosis was then securely strapped to the lever arm thereby maintaining the elbow in 90° and forearm in neutral throughout the testing session. Individuals were also securely strapped to the chair over both shoulders and at the waist in order minimize the demand on proximal segments during task performance and to restrict motion in other planes [50]. The resolution of the signal representing the isometric torque output of the elbow flexors was scaled to 39.1 mV/ft-lb. (Biodex Researchers Tool Kit Software), sampled at 625 Hz and smoothed over a 10-pt median epoch (MP150, BioPac Systems, Inc.). This processed signal was provided as visual feedback representing the subject’s elbow flexor force output in ft-lbs (this unit of measurement provided a meaningful frame of reference for the subject) and displayed on a 14-inch computer monitor located within 1 meter in front of the subject. To create a 0 value baseline indicating that the arm was indeed relaxed between contractions, the extension torque value created by the weight of the relaxed arm strapped to the torque motor lever arm was added as a constant to the smoothed torque signal. Arm weight was confirmed at the start of the second session to ensure that the mechanical demands between testing sessions were identical.

### Strength Testing of the Elbow Flexors

Elbow flexion strength was defined as the MVC value of the elbow flexors. The MVC was calculated as the difference between the maximum value and the 0 value resting baseline. Subjects performed a minimum of three isometric MVCs against the stationary lever arm at the start of each testing session and then one final maximal contraction at task failure for each fatiguing contraction. To establish the baseline MVC, subjects were instructed to gradually increase their elbow flexion force to maximum over 3-sec and then hold that maximum force for 3-sec before relaxing. Standard verbal encouragement was provided [2] throughout the contraction and subjects were given visual feedback of their force output on the computer monitor. There was a 1–2 minute rest period between each contraction. If the MVC trials were not within 5% of each other or if subjects produced more torque with each trial, subjects performed additional contractions. The highest torque output was used as the baseline MVC measure for each visit and was compared to the final MVC performed at task failure of the fatiguing contraction.

### Sustained Submaximal Contraction and tDCS

The fatiguing contractions were performed at a target force of 20% of the MVC measured at the start of the first test session. Therefore, the same target force was used for both testing sessions. It should be noted that the MVC force measured at the start of each session did not differ between Visit-1 and Visit-2 (171.47±57.71 vs. 168.91±57.06 N⋅m, *P*>0.05). Visual feedback of the force output was presented as a horizontal line across the computer screen whose position would shift up or down based upon the force output from the elbow flexors by having the horizontal time display set to 100 msec epochs and the vertical gain for force output set to 2%MVC/cm. The subject was instructed to keep the force output line as close to the target force line located in the middle of the screen for as long as possible. Task failure was declared when the feedback line drifted below 50% of the target force (i.e. ±10% MVC equivalent to ±5 cm on the screen) for longer than 3 seconds. Verbal encouragement was provided to subjects to restore the position of the feedback line if they drifted away from the target force. The same experimenter (PSW) monitored the subject’s arm and body position and provided verbal feedback to correct any shifts in upper arm position on the elbow support. At the end of the fatigue task, but prior to relaxing, the subjects were instructed to contract maximally and hold it for 2–3 seconds.

Anodal or sham tDCS was delivered to the right motor cortex, opposite of the left non-dominant arm, using a constant current stimulator (NeuroConn Eldith I Channel DC Stimulator Plus, Rogue Resolutions, Cardiff, United Kingdom). For both conditions, two conductive-rubber electrodes (35 cm^2^) each enclosed in sponge pouches soaked with normal 0.9% saline solution (McKesson USP Normal Saline Sterile 0.9%, McKesson Medical-Surgical, Richmond, VA) were placed on the subject’s pre-moistened scalp with the stimulating electrode on the right motor cortex centered over the hotspot for the biceps brachii (identified *via* TMS see below) and the active reference electrode on the left forehead just above the left eyebrow/orbit. During the anodal stimulation condition, a continuous current (1.5 mA) was delivered (35 cm^2^ electrodes) for a current density of 0.043 mA/cm^2^. The maximum duration for current delivery was 20 minutes with an 8-second ramp at the start and end. For the sham stimulation condition, current was delivered for just 30 seconds at the start and then the device stopped the current.

### Electrical Recordings

Voluntary and evoked electromyographic (EMG) signals were recorded from the left biceps brachii and brachioradialis muscles using bipolar surface electrodes (Ag-AgCl, 8-mm diameter, interelectrode distance 25-mm, Trace 1, Nikomed, Huntingdon Valley, PA) longitudinally over the two muscle bellies on shaved, abraded and cleaned skin with the reference electrode placed on the medial epidcondyle. EMG signals were amplified (1,000x), band-pass filtered (10–500 Hz), and sampled at 2,500 Hz using a 16-bit data acquisition system (MP150, BioPac Systems Inc., Goleta, CA).

### Transcranial Magnetic Stimulation

**Figure 2:**
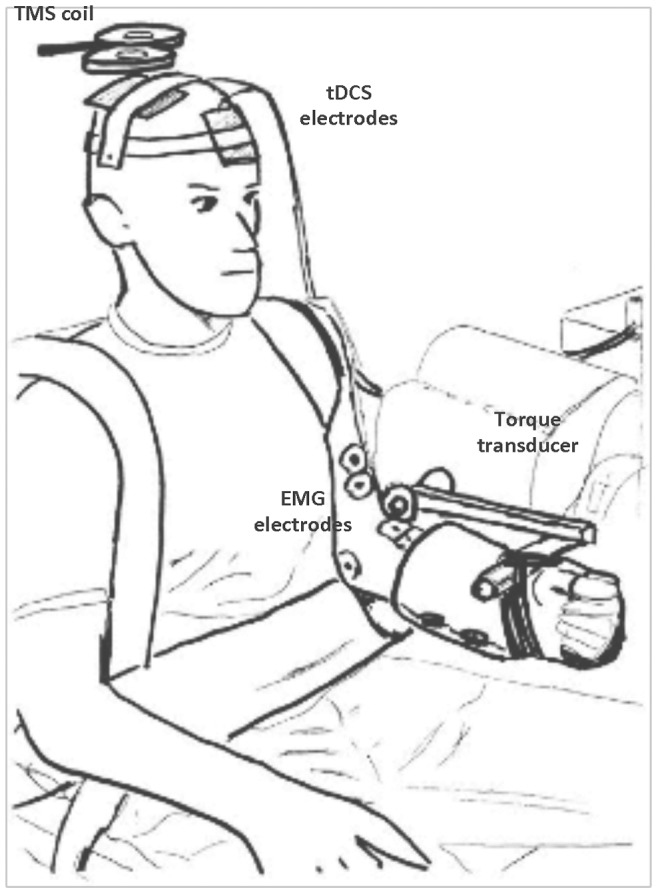
Experiment setup and subject positioning. Subjects were seated in an adjustable chair with the left elbow flexed to 90° and the forearm in neutral. With upper arm supported under the elbow, the wrist/hand were immobilized in an orthosis strapped to the adjustable length lever arm of the torque transducer. Transcranial direct current (tDCS) electrodes, placed over the right motor cortex and above the left orbit, were secured under straps.

Single pulse TMS was used to find the optimal location to place the tDCS electrodes and to evaluate the effect of tDCS on measures of corticospinal excitability during the fatiguing contraction. A hand-held 70-mm diameter figure-of-8 focal coil connected to a BiStim^2^ stimulator (Jali Medical Inc., Woburn, MA) attached to two Magstim 200^2^ stimulators (The Magstim Co. Ltd., Whitland, United Kingdom) was used to deliver a monophasic magnetic pulse. The coil was positioned tangentially to the lateral surface of the scalp in the region of the right motor cortex with the handle pointing backwards and laterally 45° from midline. With the coil is this location and position; current is expected to flow from a posterior-lateral to anterior-medial direction in the cortex [51], [52]. To find the optimal location that consistently elicited the largest peak-to-peak amplitude MEP in the biceps (i.e., the motor hotspot), suprathreshold single TMS pulses (∼60–90% of stimulator output) were delivered while subjects performed short duration (2–5 sec) submaximal isometric contractions at 20% MVC. Subjects received the same visual feedback as they would during the fatiguing contraction. The coil was systematically moved in 1-cm increments around the anatomical location on the scalp corresponding to the upper extremity distribution in the underlying brain based upon EEG 10/20 measurements [21]. After identifying the biceps motor hotspot, the two tDCS sponge electrodes were placed on the subject’s scalp (see above and **Figure 2**) so that from this point forward, all TMS pulses were delivered through the tDCS electrode located over the right motor cortex. The motor hotspot position was confirmed and marked on the tDCS sponge for consistent coil placement throughout the fatiguing contraction. To determine active motor threshold (AMT), subjects performed 4 of the short duration 20% MVC contractions during which a single pulse was delivered while the subject maintained the force output at the target force line before and after the TMS pulse. The maximum EMG peak-to-peak amplitude was quantified across the 500 milliseconds prior to the stimulus artifact and averaged across the 4 trials. AMT was defined as the minimum stimulator intensity, reported as percent of stimulator output (%SO), that evoked an MEP with a peak-to-peak amplitude twice the amplitude of the averaged EMG baseline in at least 50% of the MEPs [53], [54]. The stimulator intensity was then varied by 3–5% to confirm AMT. To evaluate corticospinal excitability during the fatiguing contraction, the TMS stimulus intensity was increased to 130% of the AMT. AMT was not significantly different between sessions (AMT mean Visit-1∶63±16%SO, range: 40–100%SO; AMT mean Visit-2∶62±16%SO, range: 35–100%SO; ICC_(3,1)_ = 0.927); therefore, the test stimulus intensity calculated for Visit-1 was also used during Visit-2 (test stimulus intensity mean: 78±15%SO, range: 52–100%SO).

### Data Analysis

Data were analyzed offline using AcqKnowledge software package version 4.2.0 (Biopac Systems Inc., Goleta, CA). The TTF was measured from the recorded torque output signal starting from when the torque signal reached the target force and ending at the onset of the final MVC. MVC values were converted from ft-lbs to N⋅m for analysis. The peak-to-peak amplitudes for the 6 evoked MEPs were measured from the EMG signals for the biceps and the brachioradialis and averaged together for each muscle at minute-1∶00 (baseline MEP) and minute-8∶30 (tDCS MEP).

### Statistical Analysis

SPSS version 20 for Mac (SPSS Inc, Chicago, IL) was used for the statistical analyses and an α of 0.05 was required for statistical significance. Data are reported with mean±SD in the text and mean±SE in the figures, and when appropriate, effect sizes (ES: partial η^2^) are provided. Paired *t-*tests were used to compare TTF between stimulation conditions. For data with serial observations, repeated measures analysis of variance (RM-ANOVA) procedures were performed for within subject’s factors for StimCondition (2-levels: anodal tDCS vs. sham tDCS) and Time (2 levels: Pre-Stim vs. Post-Stim; minute-1∶00 vs. minute-8∶30; 4 levels: Baseline, minute-1∶00, minute-8∶30, Task Failure). Post-hoc testing was used to investigate main effects with Sidak correction for multiple comparisons.

### Pilot Experiment (Figure 3)

**Figure 3:**
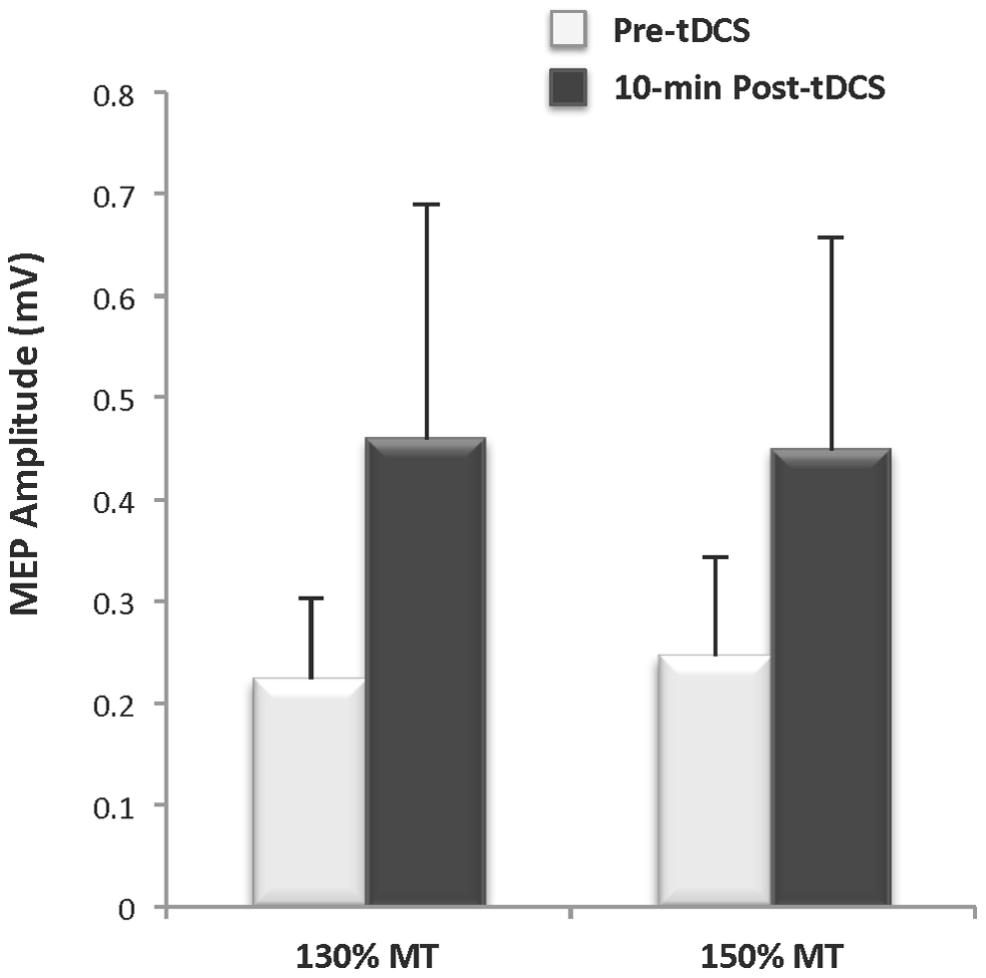
Pilot study: after-stimulation effects of anodal tDCS on cortical excitability (n = 4). Motor evoked potential (MEP) amplitude measured in the biceps 10 minutes after anodal transcranial direct current stimulation (tDCS: 1.0 mA, 35 cm^2^, 20 minutes) increased by 105% and 82% relative to pre-stimulation baseline at transcranial magnetic stimulation (TMS) intensities of 130% of motor threshold (MT) and 150% of MT, respectively (ES = 0.24).

Prior to the main experiment, we conducted a pilot experiment with 4 subjects (3 men, 1 woman) to ensure that the anodal tDCS stimulation protocol as performed in this study would, with the subject relaxed, produce the anticipated after-effects on cortical excitability as reviewed in the introduction. It was also important to determine if the changes could be successfully detected in the biceps muscle as the vast majority of studies have used the distal hand muscles to explore the effects of tDCS [32], [38]. Therefore, we used TMS as we have previously described to elicit MEPs [53], [54]. Specifically, single pulse MEPs were evoked in the biceps brachii using TMS stimulus intensities of 130 and 150% of motor threshold before and after 20-min of anodal tDCS (1.5 mA, 35 cm^2^ electrodes) with the subject’s arm relaxed. On average, MEP amplitude was nearly twice as large at 10-minutes following anodal tDCS compared to baseline (ES = 0.24) (**Figure 3**). These data confirmed that the anodal tDCS protocol used in this study can increase cortical excitability with the subject relaxed, consistent with the literature, and that the change in MEP amplitude can be successfully recorded from the biceps [43], [55]–[57].

## Results

### Neuromuscular Performance (Figures 4, 5, and 6)

**Figure 4:**
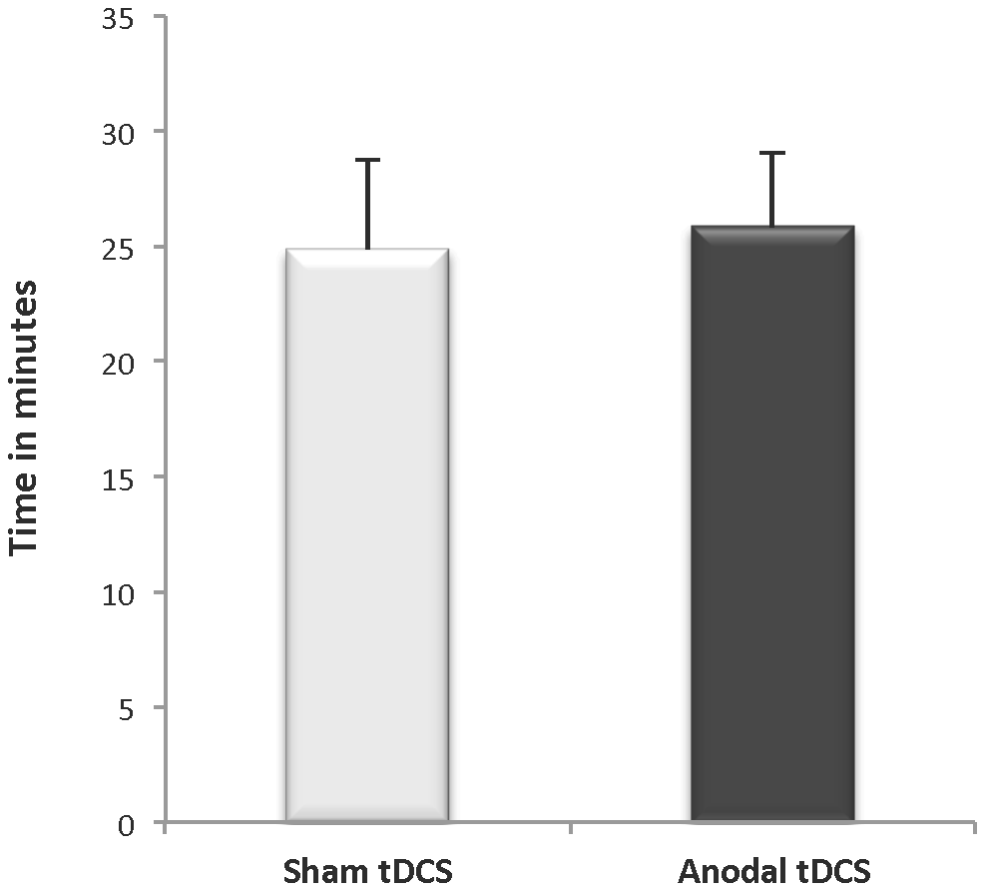
Time to Task Failure for a sustained contraction performed with anodal tDCS delivered for 20-mins and with a sham condition (n = 18). Mean (± SE) time to task failure (TTF) for sustained, submaximal elbow flexor contraction did not differ between stimulation conditions (Sham TTF 25.85±13.78 min; Anodal TTF 24.85±16.77 min; *p* = 0.639).

**Figure 5:**
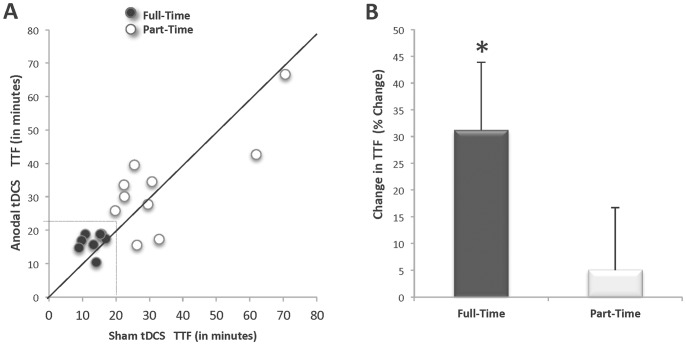
Anodal tDCS prolongs the time to task failure in subjects who received the stimulation through task failure (i.e., those who reached task failure before the anodal tDCS was discontinued). A. Individual time to task failure, B. Group percent change in time to task failure. A. *Full-Time Group* (filled circles): subjects for whom transcranial direct current stimulation (tDCS) was delivered through task failure in both stimulation conditions (n = 8). *Part-Time Group* (open circles): subjects for whom the tDCS terminated before they reached task failure for one or both stimulation conditions (n = 10). *Solid line* designates equivalent time to task failure (TTF) for both conditions. *Dashed lines* signify maximum tDCS stimulation duration. Data points above the solid line indicate that the TTF for the Anodal tDCS stimulation condition was longer than the TTF for the Sham condition. Seven of the 8 Full-Time subjects had a longer TTF with Anodal tDCS. An equal number of Part-Time subjects (n = 5 and n = 5) are on both sides of the equivalence line. B. Percent change in TTF with anodal stimulation by group. Mean percent change in time to task failure increased for the Full-Time group with Anodal tDCS but not for the Part-Time group (*p<.05).

There was no systematic effect of the anodal tDCS stimulation for altering the TTF for the entire subject set (n = 18; *p* = 0.64 ES = 0.01) (**Figure 4**). Recall, that because of the safety recommendations regarding the duration for tDCS delivery, the maximum amount of time a subject received tDCS stimulation was 20 minutes, which meant that many subjects (n = 10) were able to perform the fatigue task for a time that exceeded the 20 minutes that the tDCS was delivered. Accordingly, subjects were categorized *a posteriori* into two tDCS-time groups: *Full-Time* (n = 8, 2 women and 6 men) and *Part-Time* (n = 10, 7 women and 3 men). The Full-Time group included subjects whose TTF, for both testing sessions, occurred prior to the termination of the tDCS (i.e. they received tDCS throughout the entire fatigue task). The Part-Time group included subjects whose TTF, for one or both testing sessions, extended beyond the termination of the tDCS (i.e. they did not receive tDCS throughout the entire fatigue task). **Figure 5A** illustrates the individual subject TTF response by stimulation condition. Note that 7 of the 8 subjects in the Full-Time group experienced an increase in the TTF during the anodal tDCS condition. An analysis of these sub-groups indicated that the Full-Time group exhibited a 31% longer TTF with anodal tDCS stimulation when compared to the sham stimulation condition (16.48±2.87 min vs. 13.13±1.34 minutes, *p = *0.04, ES = 0.47) (**Figure 5B**). Conversely, the Part-Time group did not exhibit a differential effect in TTF between stimulation conditions (Anodal tDCS TTF: 33.34±14.56 min vs. Sham tDCS TTF: 34.23±17.46 min, *p* = 0.81, ES = 0.01). An examination of the visit order for the subset of subjects in the Full-Time group revealed that 63% of this subset (5 of 8) performed the anodal tDCS condition during Visit-1, whereas 37% (3 of 8) performed the sham tDCS during Visit-1.

**Figure 6:**
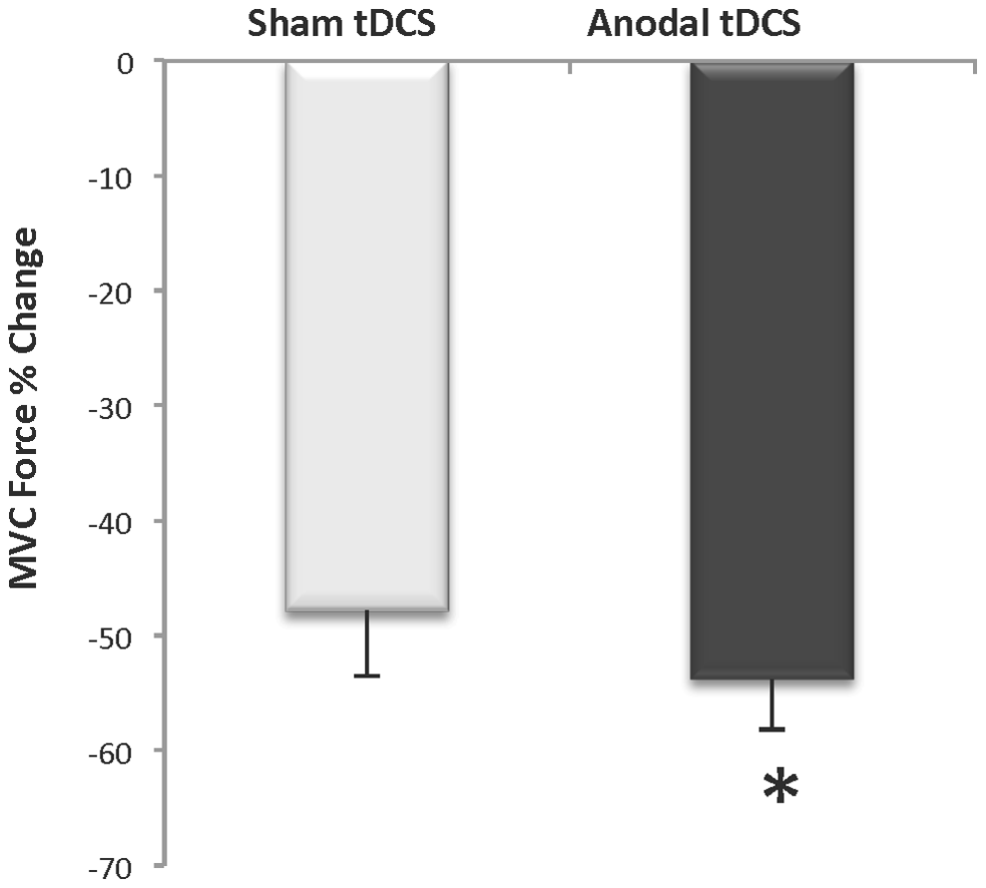
Percent decline in maximum voluntary contraction force for the Full-Time group (n = 8). Mean decline in MVC force was 6% greater after task failure in the Anodal stimulation condition compared to the Sham stimulation condition (*p<0.05).

At the end of the fatigue task subjects were asked to perform an MVC and the percent decline from baseline MVC was calculated. Interestingly, the Full-Time group had a greater decline in MVC force in the anodal tDCS condition compared to the sham tDCS session (53.8±12.5 vs. 47.9±16.1%; StimCondition X Time Interaction: *p* = 0.05, ES = 0.44) (**Figure 6**). The Part-Time group exhibited a similar level of decline in MVC in both conditions (Anodal tDCS: 67.7±18.4% vs. Sham tDCS: 72.1±23.7%, StimCondition X Time interaction p = 0.56, ES = 0.01). It is also important to note that there was no significant difference in the mean target force exerted by both groups during fatigue-task performance (Full-Time: 11.44±3.76 vs. Part-Time: 9.38±3.13 N⋅m; *p* = 0.23).

The remainder of the results will be limited to the data derived from the Full-Time group only.

### Rating of Perceived Exertion (Figure 7)

**Figure 7:**
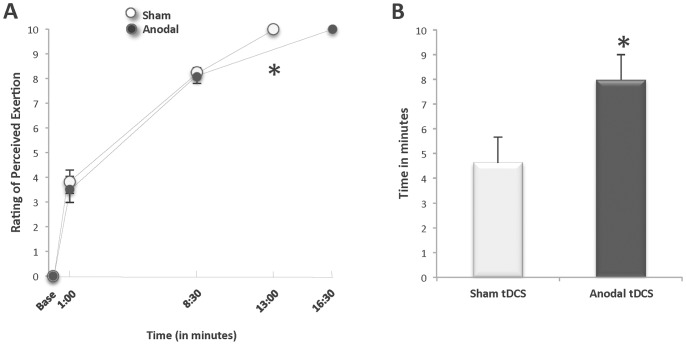
Effects of anodal tDCS on rating of perceived exertion during sustained contractions. A. Rating of perceived exertion (RPE), B. Amount of time spent at RPE of 8–10 (n = 8). A. Mean RPE was the same before (baseline), during (minute-1∶00 and minute-8∶30), and at the mean time to task failure (TTF) of the fatiguing contraction for both the Anodal and Sham tDCS conditions. From baseline to minute-8∶30, the mean increase in RPE over time did not differ between the two stimulation conditions. Between minute-8∶30 and the mean TTF, the rate of change for RPE was significantly slower during Anodal tDCS condition than the Sham tDCS condition (*p = 0.03). B. Subjects sustained their contraction time while at an RPE level between 8 and 10 for an average of 8.0±2.9 minutes with anodal stimulation compared to 4.6±2.9 minutes with sham stimulation (*p = 0.04).

There were no differences in the values for RPE between stimulation conditions for the Full-Time group prior to starting the fatigue task, at minute-1∶00, minute-8∶30 and at task failure (Anodal tDCS 0.0±0.0, 3.5±1.5, 8.1±0.8, 10.0±0.0 vs. Sham tDCS: 0.0±0.0, 3.8±1.3, 8.3±0.5, 10.0±0.0, StimCondition X Time Interaction p = 0.78) with all subjects at their maximum level of exertion (i.e., RPE 10 = “extremely hard”) at task failure in both stimulation conditions (**Figure 7A**). It is important to note that because the anodal stimulation increased TTF by a mean of 30% (equivalent to 3∶21 min), the absolute time points when RPE was assessed occurred at different relative time points to the total TTF in each condition; therefore, minute-8∶30 occurred earlier at 53±12% of TTF for the anodal condition and later at 68±17% of TTF for sham (% of TTF: StimCondition x Time Interaction and StimCondition Main Effect *p* = .08 ES = 0.37). The mean rate of change in RPE between minute-8∶30 and task failure was significantly slower for the anodal condition (Slope: RPE/Time between 8∶30 and Task Failure: Sham 0.38±0.11, Anodal 0.24±0.1; *p* = 0.03) such that after 8∶30 minutes, it took significantly longer time to reach the same RPE of 10 at task failure with anodal tDCS (**Figure 7A**). Therefore, the subjects in the Full-Time group continued the contraction at an RPE of 8–10 for a mean of 8.0-min with anodal stimulation compared to 4.6-min with sham stimulation (Sham tDCS time at 8–10 RPE: 4.6±2.9 min; Anodal tDCS time at 8–10 RPE: 8.0±2.9 min; *p* = 0.04 ES 0.47) (**Figure 7B**).

### Motor Evoked Potential Amplitude (Figure 8)

The data for 7 of the 8 subjects in the Full-Time group were analyzed because MEPs could not be reliably obtained in one subject during the first test session due to equipment failure. StimCondition x Time Interactions were not observed for changes in MEP amplitude for either the biceps brachii or brachioradialis muscles (StimCondition x Time Interaction: biceps *p* = 0.27 and brachioradialis *p = *0.90). As expected, a Time Main Effect was observed for both muscles indicating an increase in MEP amplitude as the fatiguing contraction progressed (Time Main Effect: biceps: *p* = 0.02 and brachioradialis: *p* = 0.00). A StimCondition Main Effect was observed for the biceps brachii (StimCondition Main Effect: biceps *p* = 0.04), as the MEP amplitudes prior to applying the anodal stimulation as well as after 7-min of delivering the anodal stimulation were significantly lower than those evoked during the sham condition (*p* = 0.05), although the percent change in amplitude between the two conditions was not significantly different (*p* = 0.56). No such stimulation condition main effect was observed for the brachioradialis (Stim Main Effect: brachioradialis *p* = 0.31).

**Figure 8:**
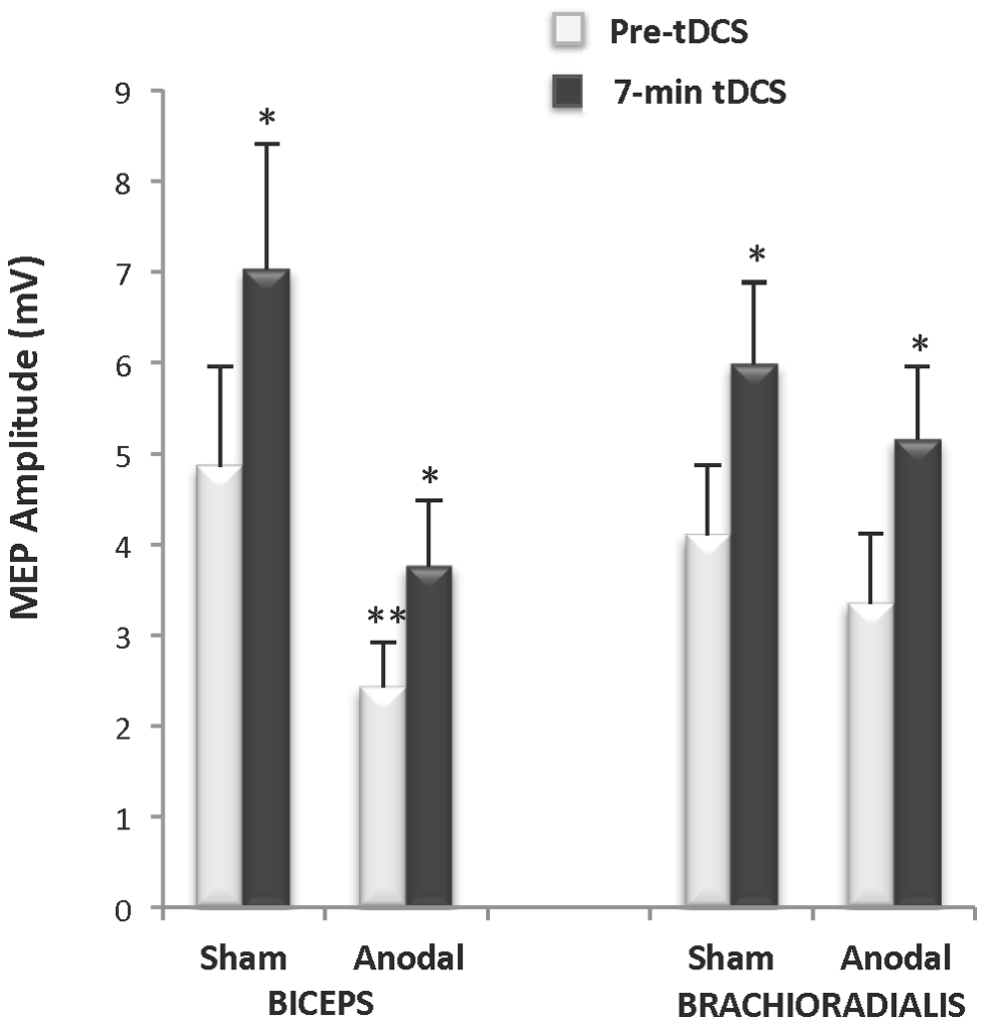
During-stimulation effects of tDCS and fatiguing contraction on cortical excitability for the Full-Time group (n = 7). Motor evoked potential (MEP) amplitude was obtained in 7 of 8 subjects in the Full-Time group. Simulation condition x Time interactions were not observed for changes in MEP amplitude for either the biceps brachii or brachioradialis muscles (*p = *0.90). MEP amplitude increased in both muscles between the two times points in both muscles (*biceps: *p* = 0.02 and brachioradialis: *p* = 0.00). A Stimulation Condition main effect was observed for the biceps brachii (**p = 0.04), as the MEP amplitudes prior to applying the anodal stimulation as well as after 7-min of delivering the anodal stimulation were significantly lower than those evoked during the sham condition (*p* = 0.05), although the percent change in amplitude between the two conditions was not significantly different (*p* = 0.56). No stimulation condition main effect was observed for the brachioradialis (*p* = 0.31).

## Discussion

The purpose of this study was to determine if excitatory brain stimulation with anodal tDCS delivered *while performing* a sustained submaximal contraction (i.e. 20% MVC) could increase the duration that the contraction could be sustained. In agreement with the hypothesis, in individuals whose TTF occurred prior to the termination of the anodal stimulation protocol (i.e., Full-Time group), anodal brain stimulation dramatically increased the TTF by more than 30%, increased the amount of muscle fatigue by 6%, and prolonged the period of time that the subjects could sustain a high degree of effort (i.e. contract at an RPE between 8–10) by 38%. It should be noted that this enhancement in the TTF and associated force decline was not observed in the individuals whose TTF *exceeded* the anodal brain stimulation duration (i.e., Part-Time group). The findings for the Part-Time group raise the question of whether the effects of the tDCS on muscle performance are only available if the stimulation remains active during the contraction or whether the effects are only beneficial for individuals with comparatively low endurance capacity. Based on the current data it is not possible to conclusively answer this question, and further work is required to more fully understand the mechanisms of tDCS on the changes in TTF and muscle fatigue. However, the novel findings reported for the Full-Time group when compared to the Part-Time group do suggest that 1) motor cortical excitability plays a crucial mechanistic role in the TTF of sustained submaximal fatiguing contractions, and 2) that anodal brain stimulation has the potential to enhance human muscle endurance, which has obvious implications for rehabilitation medicine. Below, these findings are discussed in further detail.

The observation that the delivery of a neuromodulatory protocol designed to increase motor cortical excitability (i.e., anodal tDCS) prolonged the TTF of a sustained submaximal contraction as well as increased the amount of muscle fatigue measured at task failure in the subset of individuals who received the stimulation throughout their entire fatigue task indicates that supraspinal mechanisms are mechanistically involved in task failure of fatiguing contractions. While these changes in performance suggest that the anodal brain stimulation increased intracortical excitability in the Full-Time group, the actual direct measures of corticospinal excitability taken during the contraction with brain stimulation did not show a change in MEP amplitude relative to the sham condition after 7 minutes of tDCS. That we also observed that the anodal tDCS slowed the rate of increase in perceived effort during the last half of the fatiguing contraction such that the subjects in the Full-Time group were able to work at a high level of effort 38% longer than without the anodal tDCS suggests that the RPE data may provide some insight into the mechanisms of tDCS behind the increases in task duration and muscle fatigue in the Full-Time group as well as provide an explanation for the lack of effect in the Part-Time group.

During the performance of a sustained submaximal contraction, the level of force output remains the same but the sense of effort and the amount of neural drive progressively increases as fatigue develops [15], [58]. Although RPE is subjective, it is attributed to the corollary discharge or the replica of the central motor command for the muscle that is instead sent to the sensory regions and thus the value for RPE is thought to reflect the amount of neural drive needed to perform the task [1], [2], [59]. That the task duration at which the Full-Time subjects were able to sustain the contraction while perceiving a high degree of effort increased by 38% suggests that the anodal tDCS was able to provide the additional excitatory input to the motor cortex needed when task failure was eminent. Therefore, in order to overcome the continual decline in spinal excitability, it seems plausible that the anodal tDCS added enough excitability to the already high degree of volitional neural drive exerted by the subjects to essentially provide a “boost” when it was most needed potentially through enhanced motivation and/or descending drive to the motorneuron pool thereby prolonging contraction time at a high degree of effort [7]. This conclusion is further supported by the increase in muscle fatigue measured at task failure (i.e., greater reduction in MVC at task failure), which suggests that the excitatory input provided by the anodal tDCS increased task duration most likely through the recruitment of additional motor units in the spinal cord (see also the companion paper). The finding that the individuals in the Part-Time group did not have a change in task duration or amount of muscle fatigue at task failure suggests that by the time that the subjects in the Part-Time group needed the assistance from the anodal tDCS to overcome declining spinal excitability (i.e. when their RPE was at an 8 or higher), the stimulation had already stopped. Perhaps when subjects were working at an RPE level less than 8, the effect of the stimulation may have been negligible as the amount of volitional drive was sufficient to continue to activate the spinal motor pool necessary for task performance.

The second purpose of the study was to use single pulse TMS to explore the relationship between changes in cortical excitability and performance. While it is a common experimental approach to evoke MEPs during performance of a fatiguing contraction [2], [6], [11], [13], [60], [61], to our knowledge this is the first study to evoke MEPs during the delivery of tDCS concurrent with the performance of a fatiguing muscle contraction. The lack of difference in MEP amplitude between the two stimulation conditions and between the two groups may be explained by when the measures were taken during the experiment or to a “ceiling-effect” in membrane excitability. In light of the RPE data discussed above, changes in cortical excitability may have been missed as no measures were taken after minute-8∶30; therefore, it is not possible to determine if cortical excitability changed in relationship to the performance measures. Measuring cortical excitability during stimulation after a period of time found sufficient to induce short-term after-effects [35], [41] would be expected to show an increase in MEP amplitude relative to the sham condition; however, physiologically speaking, increases to neural excitability cannot be unlimited [26]. In this study there were two sources of excitability to the nervous system: the sustained submaximal contraction and the tDCS. MEP amplitude has been shown to progressively increase as fatigue develops during sustained submaximal contractions [2], [3], [60], [62] a finding demonstrated in this study for both conditions and for both subsets. Therefore, a lack of difference between stimulation conditions may be due to a “ceiling-effect” in membrane excitability reached by the already high background level of excitation from the contraction [30], [31]. It may be more significant that there was not a decrease in MEP amplitude due to the mechanisms of homeostatic plasticity where the addition of external excitatory stimulation to the high level of background excitation could be expected to cause a rebound inhibitory effect to keep neuronal modulation within a a physiologic range [19], [31], [63]. Finally, even though the effects of tDCS have been found to be primarily intracortical [27], [28], [30], [64], it is worth questioning whether the tDCS increased descending drive to the motorneurons thereby minimizing the need to increase cortical excitability to compensate for motorneuron resistance [15] (See also companion paper). Therefore, while not conclusive, the during-stimulation TMS results of this study do not exclude the possibility that the anodal tDCS modulated cortical excitability during the fatiguing contraction in the Full-Time Group.

The key finding in this study that the addition of anodal brain stimulation provided throughout the entire performance of a sustained submaximal contraction increased muscular performance suggests that tDCS can increase exercise capacity. Specifically the observations in the Full-Time subset that anodal tDCS received throughout the contraction time increased TTF, increased muscle fatigue, and also prolonged the period of time that healthy individuals who were low to moderately active could sustain a high degree of effort, indicate that tDCS facilitated the type of exercise performance required to derive the classical benefits from exercise training. Exercise, as distinguished from motor training, is defined as physical activity done with the purpose of improving or sustaining components of health and physical fitness such as muscular strength, endurance, and power [65]. To achieve improvements, the exercise demands must sufficiently overload or stress the neuromuscular system during the training session beyond that typically confronted in daily life [65], [66]. Thus in order to successfully overload a muscle to increase endurance, the muscle needs to work for a longer period of time during the exercise session and experience a greater level of fatigue at the end of the session [67]. It would be both naïve and presumptuous to assume that the findings of this study– which suggest that anodal tDCS can enhance exercise capacity of a single task in a single session in the subset of healthy individuals who received the stimulation throughout the contraction time– can be generalized to clinical populations, other muscle groups and motor tasks, or used to predict a response to repetitive training sessions. However, these stated limitations do most certainly suggest rich opportunities for future study along these lines.

## Conclusion

The novel finding that the targeted delivery of non-invasive brain stimulation known to increase excitability to supraspinal structures while performing a sustained submaximal contraction improved the TTF in the subset of subjects (n = 8) who received anodal tDCS throughout the contraction duration suggests that changes in supraspinal excitability are mechanistically involved in neuromuscular fatigue. To date the primary clinical application of tDCS to modulate cortical excitability in studies that explore motor function has been to facilitate motor control and learning as measured by a change in speed and accuracy of performance as well as long term retention of performance changes [19], [25], [33], [37], [55], [68]. Therefore, this finding also indicates that tDCS can become as useful a tool to explore the neural mechanisms of fatigue as it has become for understanding the neuroplastic mechanisms of motor learning. Additionally, our finding that when anodal tDCS was received throughout the performance of a fatiguing submaximal isometric contraction endurance exercise capacity increased, as measured by the ability to continue to contract for a longer period of time at a high level of perceived effort resulting in a greater amount of muscle fatigue at task failure, may indicate tremendous clinical utility of tDCS in neurologic rehabilitation and physical therapy practice. Future studies applying these results in patient populations will help to determine if tDCS will be as useful in enhancing exercise capacity in individuals both with and without fatigue as it is for improving motor skill performance and motor learning. Clearly ongoing study is needed in both applied neurorehabilitation practice to assess the therapeutic benefits of tDCS as well as mechanistic neurophysiologic research to better understand how to target non-invasive brain stimulation techniques in order to optimize neural adaptations that underlie the changes in multiple facets of motor function.

### Limitations

It is important to recognize that the interpretation of the effects of tDCS on task duration of sustained submaximal contractions are based upon a subset of subjects (n = 8) whose contraction time ended prior to the cessation of the stimulation. While an *a priori* decision to group the subjects into a *full-time* and *part-time* group based upon contraction duration would have been optimal, as this was the first study to investigate the effect of tDCS when delivered simultaneously with the contraction, as opposed to a preconditioning treatment as previously published, these results were not what we could have been anticipated. Therefore, the *a posteriori* analysis performed was an appropriate method to adopt based upon the data from the study.

## Global Conclusion of the Collective Findings from the Companion Papers

This paper, along with the preceding companion paper, have collectively sought to better define and delineate the contribution that supraspinal circuits have in determining the TTF of sustained submaximal contractions. In the first paper task failure occurred after a similar mean decline in motorneuron excitability developed coupled with a similar mean increase in corticospinal excitability. During task performance, as the amount of intracortical inhibition dropped, the amount of intracortical facilitation and upstream excitation of the motor cortex remained unchanged. The findings from that experiment suggest that the motor cortex is able to compensate for changes in spinal excitability until a critical amount of change in both regions develops, which implies that unless more drive is provided to the motor cortex to sustain or strengthen descending drive, failure occurs. In this paper, the application of anodal tDCS during the performance of a fatigue task prolonged task duration, increased the amount of muscle fatigue and the amount of time subjects could exert a high amount of effort. These results suggest that the anodal tDCS, when delivered throughout the duration of the contraction, provided the additional excitatory input to the motor cortex needed when task failure was eminent in order to overcome the increase in spinal resistance that could not otherwise be met by innate volitional drive. Collectively, the results from these companion papers provide complimentary evidence to support the conclusion that the capacity of supraspinal inputs to endlessly override the decline in spinal motorneuron excitability is eventually limited by the failure of upstream drive delivered to the motor cortex and not the development of intracortical inhibition.
